# *Klebsiella pneumoniae* Co-Producing NDM-5 and OXA-181 Carbapenemases, South Korea

**DOI:** 10.3201/eid2106.150048

**Published:** 2015-06

**Authors:** Sun Young Cho, Hee Jae Huh, Jin Yang Baek, Na Yeon Chung, Jae Geum Ryu, Chang-Seok Ki, Doo Ryeon Chung, Nam Yong Lee, Jae-Hoon Song

**Affiliations:** Samsung Medical Center, Sungkyunkwan University School of Medicine, Seoul, South Korea (S.Y. Cho, H.J. Huh, N.Y. Chung, J.G. Ryu, C.-S. Ki, D.R. Chung, N.Y. Lee, J.-H. Song);; Asia Pacific Foundation for Infectious Diseases, Seoul (J.Y. Baek, D.R. Chung, J.-H. Song)

**Keywords:** *Klebsiella pneumoniae*, carbapenemase, Enterobacteriaceae, South Korea, antimicrobial resistance, bacteria, New Delhi metallo-β-lactamase, NDM-5, oxacillinase 181

**To the Editor:** Carbapenemase-producing *Enterobacteriaceae* are being reported worldwide. Travel, medical tourism, and cross-border transfer of patients might play a role in the spread of these bacteria (*1**,**2*). *Klebsiella pneumoniae* co-producing New Delhi metallo-β-lactamase 5 (NDM-5) and oxacillinase 181 (OXA-181) carbapenemases was detected in South Korea in 2014.

On April 13, a 75-year-old man who had had a cerebral infarction was transferred from a tertiary care hospital in Abu Dhabi, United Arab Emirates (UAE), to Samsung Medical Center (Seoul, South Korea) for rehabilitation therapy. In Abu Dhabi, he had received broad-spectrum antimicrobial drugs for aspiration pneumonia. While at Samsung Medical Center, he experienced septic shock and acute respiratory failure due to pneumonia and was transferred to the medical intensive care unit (ICU). Carbapenem-resistant *K. pneumoniae* (strain CC1409-1) was isolated from a culture of bronchoalveolar lavage fluid. He was given meropenem and colistin for treatment of pneumonia, was discharged, and returned to the UAE.

Four months later, carbapenem-resistant *K. pneumoniae* (strain CC1410-1) was identified in the tracheal aspirate of a 74-year-old woman admitted to the surgical ICU at Samsung Medical Center for traumatic intracranial hemorrhage. She had no underlying disease or previous history of hospitalization or travel abroad. She was given colistin and piperacillin/tazobactam. Following the identification of colistin resistance, colistin was switched to tigecycline. However, her clinical condition worsened (aggravated pneumonia), and she died of refractory respiratory failure.

In vitro antimicrobial drug susceptibility tests of 2 isolates were performed by using broth microdilution. Results were interpreted following Clinical and Laboratory Standards Institute guidelines (*3*), except for those for colistin and tigecycline, for which European Committee on Antimicrobial Susceptibility Testing breakpoints were used (*4*). The first isolate was susceptible to colistin but none of the other antimicrobial agents tested (cefepime, ceftriaxone, ceftazidime, aztreonam, amikacin, ciprofloxacin, trimethoprim/sulfamethoxazole, ertapenem, imipenem, and meropenem)whereas the second isolate was susceptible only to tigecycline. Modified Hodge tests for both isolates showed positive results. Production of metallo-β-lactamase was detected by an imipenem-EDTA double-disk synergy test.

The presence of carbapenemase genes was determined by PCR and DNA sequencing (*2*). The *bla*_NDM_ and *bla*_OXA-48_ genes were detected in both isolates. The PCR product sequences were consistent with those of NDM-5 (GenBank accession no. JN104597.1) and OXA-181 (GenBank accession no. JN205800.1). Further analyses for other β-lactamases (TEM-type, SHV-type, and CTX-type) and 16S rRNA methylase aminoglycoside resistance determinants (*armA*, *rmtA*, *rmtB*, *rmtC*, *rmtD*, *rmtF*, and *npmA*) revealed that both isolates carried *bla*_TEM-1_, *bla*_SHV-11_, *bla*_CTX-M-15_, and *rmtB* genes.

Clonal relatedness was investigated by using multilocus sequence typing and pulsed-field gel electrophoresis (PFGE) (*5**,**6*). Multilocus sequence typing revealed that both isolates belonged to sequence type 147. PFGE showed that both isolates were the same strain (Figure).

**Figure:**
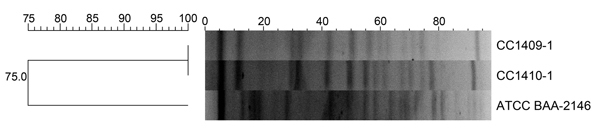
Dendrogram of pulsed-field gel electrophoresis patterns showing the genetic relationship between 2 *Klebsiella pneumoniae* isolates co-producing New Delhi metallo-β-lactamase 5 and oxacillinase 181 carbapenemases, South Korea, 2014. ATCC BAA-2146 indicates New Delhi metallo-β-lactamase 1 *K. pneumoniae* used as a reference strain. Scale bar indicates percentage genetic relatedness.

The 2 patients were never hospitalized in the same ward and there was a substantial time lag between their hospitalizations. However, given sequence type and PFGE patterns between 2 isolates, we suspected nosocomial cross-transmission and performed infection control measures, including strict contact precautions and enhanced environmental cleaning with daily monitoring in the surgical ICU. In addition, environmental cultures and active surveillance cultures (rectal swabs and respiratory samples) on all patients in the units where these isolates were identified were performed to find asymptomatic carriers or contaminated environments as potential sources of transmission. All samples tested were negative for carbapenemase-producing *Enterobacteriaceae*. No further cases were reported in the hospital.

NDM-5 was first identified in a multidrug-resistant *Escherichia coli* sequence type 648 isolate from a patient in the United Kingdom who had a recent history of hospitalization in India (*7*). NDM-5 differs from existing enzymes due to substitutions at positions 88 (Val→Leu) and 154 (Met→Leu). OXA-181, a variant of OXA-48, was initially reported in India but has been sporadically detected in the United Kingdom, the Netherlands, France, New Zealand, Oman, and Singapore (*8*). It has also been found to be associated with other carbapenemase genes, such as the *bla*_NDM-1_ and *bla*_VIM-5_ genes, and particularly in isolates with a link to the Indian subcontinent.

In the cases we describe, the first *K. pneumonia*e isolate was recovered from a patient transferred from the UAE. Recent studies suggest that the Middle East, a region with close ties to the Indian subcontinent that hosts a large expatriate population, may act as another reservoir of OXA-48 and NDM producers (*9**,**10*). The emergence of extremely drug-resistant isolates carrying multiple carbapenemase genes is of concern because of limited treatment options and the possibility of global dissemination by means of cross-border transfer. A collaborative interdisciplinary strategy, including active surveillance for high-risk patients and adequate infection control measures against spread of such highly transmissible multidrug-resistant strains in health care settings, is necessary.
